# Overindebtedness, unemployment, and poor mental health – and the role of sense of control: a population-based Swiss study

**DOI:** 10.3389/fpubh.2024.1324402

**Published:** 2024-04-22

**Authors:** Oliver Hämmig

**Affiliations:** Epidemiology, Biostatistics and Prevention Institute (EBPI), University of Zurich, Zürich, Switzerland

**Keywords:** unemployment, overindebtedness, sense of control, poor mental health, psychological distress, depression, Switzerland

## Abstract

**Background:**

Both overindebtedness and unemployment are critical life events that can result in or lead to poor mental health. What is less known is that the two partly interrelated events frequently go along with a feeling of loss or lack of control in life, which could be the main reason why they are associated with poor mental health. This has not been examined in previous research, particularly not in this combination.

**Methods:**

This study used and merged two cross-sectional data sets. Data collected in 2019 on 219 overindebted clients of the four official debt advisory centers in the Canton of Zurich were linked with a comparable subsample of 1,997 respondents from the Swiss Health Survey of 2017. The entire study population covered 2,216 adult individuals living in the Canton of Zurich.

**Results:**

The prevalence of no or low sense of control, medium to high psychological distress, and moderate to major depression was much higher among the 44 solely unemployed (36/30/12%), the 189 solely overindebted (73/83/53%), and particularly among the 30 unemployed *and* overindebted (93/97/60%) than among all 1,953 other survey participants (21/13/7%). Unemployment, overindebtedness, and a (resulting) lack or loss of control were all found to be strong risk factors for the two mental health outcomes under study. Associations, or rather negative health effects, were partly but not fully mediated by the sense of control. Overindebtedness much more strongly predicted psychological distress (*ß* = −0.37) and depression (*ß* = 0.17) than unemployment (*ß* = −0.05/0.01). The sense of control turned out to be an independent explanatory factor for poor mental health and even the strongest of all (*ß* = 0.49/−0.59).

**Conclusion:**

Improving a person’s control beliefs could be a promising measure for preventing mental health disorders in general and in people who are unemployed and/or overindebted in particular.

## Introduction

The burden of overindebtedness and the threat of unemployment, both together and independent of one another, have been found to be strongly associated with mental health, particularly poor mental health (1–11) or even suicidal behaviors (12–15). The two critical and stressful life events are closely related to each other in two different ways. For one, unemployment has been found to be a precondition of overindebtedness. Or more precisely, overindebtedness on an individual or household level can result from overspending (or overborrowing), poor income (working poverty) or income decline, family breakdown (or divorce), ill health, or—finally—from long-term or multiple unemployment or precarious employment (3, 16). For another, what overindebtedness and unemployment have in common is that they are both sources (or results) of social stigma and prejudice (17–20), and they both go along with emotional distress, feelings of powerlessness, helplessness, uncertainty about the future, and perceptions of loss of control over life (1, 11, 17).

### Empirical findings and theoretical considerations

There is an ongoing debate about the causality in the relationship between unemployment or overindebtedness and mental health (14, 21). Although overindebtedness and unemployment may reflect both a cause and a consequence of poor health or mental illness—i.e., the causal relationship may run in both directions—the research mostly finds evidence for or considers overindebtedness and unemployment as causes of poor mental health rather than consequences of poor mental health. There is substantially more evidence in the research literature for the *exposure hypothesis*, which states that unemployment (2, 4, 9, 11, 22–24) or overindebtedness (3, 5, 12, 25–28) leads to or accompanies mental illness, and less evidence supporting the *selection hypothesis*, which implies that mental illness leads to unemployment (18, 29) or overindebtedness (21).

The causal link between (longer or repeated) unemployment and (long-lasting or ongoing) overindebtedness and mental ill health seems to run through a social mechanism, mental process, and/or behavior changes: Unemployed or (over)indebted individuals face prejudice or experience discrimination by employers (19), feel shame and failure (27), or forgo medical care to avoid the expense of healthcare and medications (30). Job loss frequently leads to (over)indebtedness, and unemployment results in a loss of social networks, social status, purpose, and self-esteem (4, 19).

A study that we conducted recently found a comparably low sense of mastery or control among 219 overindebted individuals in Switzerland, which seems to be mainly responsible for their remarkably poor mental health status (1). That study indicated that the comparably and extraordinarily high prevalence of major depression and high psychological distress observed among overindebted individuals can be possibly attributed to their mostly weak mastery or low sense of control (1). In reverse, a high sense of mastery, i.e., a strong general belief of having control over one’s own life—although rarely found among the overindebted study participants—turned out to be highly protective against poor mental health (1).

Another recently published questionnaire-based study in Portugal with a self-selected non-random sample of 365 overindebted and non-overindebted consumers similarly found that overindebtedness reduces the feeling of having control over important things in life (17). The study further showed that “perceived control” partly or even fully mediates and buffers the negative outcomes and direct effects of overindebtedness on life satisfaction, self-rated health, and emotional wellbeing (17).

The findings of these two studies taken together seem to reveal the true psychological mechanism behind or the ‘missing link’ and the mostly overlooked important mediation or confounding in the strong relationship between unemployment and (subsequent) overindebtedness on the one hand and poor mental health on the other. In other words, unemployment and overindebtedness, no matter if dependent or independent events, are suspected to lead to poor mental health not directly but rather indirectly, via the perceived loss of control over events and outcomes in life that results from such incisive experiences and critical life events beyond one’s control.

The sense of mastery or control is strongly related to the psychological concepts of learned helplessness and self-efficacy, which were developed and studied by notable American psychologists Martin Seligman, Albert Bandura, and others. They are generally regarded as important factors or characteristics that strongly affect an individual’s vulnerability to depression and mental illness. Losing a job, becoming unemployed, and particularly being longer unemployed and/or losing one’s financial capability, control, and autonomy can lead to great uncertainty and a lack of prospects far into the future and, over time, to a generalized feeling of loss of control over outcomes in life. This in turn may result sooner or later in frustration, resignation, and passivity, or in learned helplessness and low self-efficacy, and finally lead to depression and mental illness. Learned helplessness is a well-known state and concept in psychology that is commonly used to explain depression and other mental disorders, such as anxiety (31, 32).

### Research questions

The aim of this study was, first, to investigate if a similarly strong negative relationship between unemployment and mental health can be observed, as was previously found for overindebtedness and mental health, in a sample of the general population with an oversampled subsample of overindebted individuals (25). Second, the study examined if these associations or relationships are substantially mediated (or confounded) by the individual characteristic of a generalized sense of mastery or control, and explored if a low sense of control in general is decisive for psychological distress and becoming depressed or developing major depression, independently of and additional to the stressful situation of overindebtedness, which was found to be a strong predictor of psychological distress and major depression (1).

*Hypothesis 1 (H1)*: Like other critical and stressful events in life beyond one’s control, losing a job and/or financial autonomy—particularly when repeatedly, continuously, or even constantly experienced—go along with an overall belief of being unable to control or change any situation in life. This is a generalized feeling of loss of control (learned helplessness).

This first hypothesis (H1) leads to the following research question:

Are both unemployment and overindebtedness, independent of each other, associated with a low sense of mastery and a perceived loss of control?

*Hypothesis 2 (H2)*: A lost, low, or non-existent feeling of control over events in life is the true cause behind the negative effects of critical life events on psychological distress and poor mental health. Unemployment and overindebtedness mainly lead to mental illness indirectly through a strongly reduced sense of control.

The second hypothesis (H2) raises the following research questions:

Are unemployment and overindebtedness equally and strongly associated with psychological distress and depression? And if yes, are these associations largely or even fully mediated or confounded by a low sense of mastery and control?

**Figure 1:**
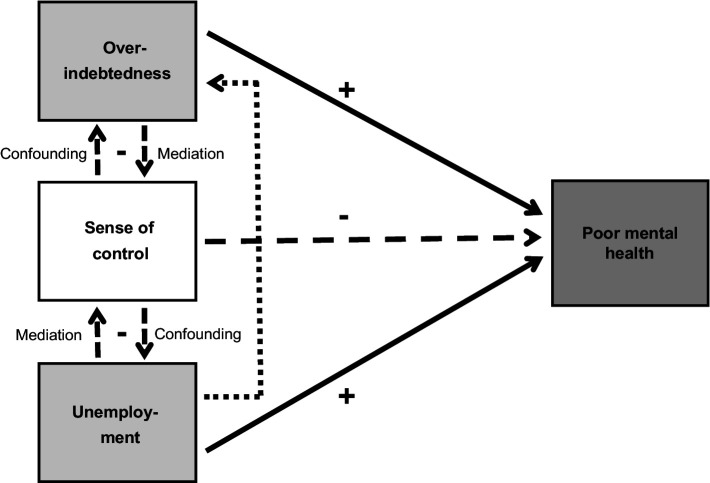
Theoretical explanatory model showing the predictor or explanatory, intermediate, and dependent variables under study and the direction of possible causal paths.

Figure 1 shows all hypothesized and studied associations, direct and indirect paths, and relationships between exposures, mediators, and outcomes.

## Methods

### Data and study population

The present cross-sectional study is based on a combined set of survey data of 219 overindebted individuals at the age of 18 years or older and 1,997 representatives of the general resident population in the Canton of Zurich of the same age. Data were collected in the year 2019 in a full sample survey among overindebted adults seeking advice from one of the four official debt advisory centers in the Canton of Zurich and then linked with a comparable subsample of respondents of the nationally representative Swiss Health Survey of 2017 of the same age and canton of residence (25). The study population covered a total of 2,216 adult individuals, and the pooled dataset was restricted to responses to a limited number of identical health-related and other questions asked in both surveys. These questions used in the overindebtedness survey of 2019 were taken from the Swiss Health Survey of 2017 for reasons of compatibility and comparability. The subsample of 219 overindebted individuals and the control group of mostly not overindebted representatives of the general population have been characterized separately and in detail in a previous study (1).

The study and survey among the 219 overindebted adult residents of the Canton of Zurich were granted exemption from requiring ethics approval, because these self-reported and completely anonymously collected survey data are not from patients and do not include medical records. As voluntary self-reports, they therefore do not fall within the scope of the Human Research Act (Kanton Zürich, Kantonale Ethikkommission; BASEC-Nr. Req-2019-00173). Similarly, no approval was required for the use of the secondary and generally accessible data of the Swiss Health Survey on randomly selected inhabitants of Switzerland; these data are periodically collected and provided by the Federal Statistical Office.

### Measures

#### Overindebtedness

Overindebtedness was measured simply by being a client of one of the four debt advisory centers of Canton Zurich and by having agreed to participate in the overindebtedness survey. In the experience of the debt advisors, already seeking advice from an official debt advisory center by definition means being overindebted or, more precisely, being hardly or no longer able to repay and/or service all debts and outstanding accounts fully and on time (1, 25). As respondents of the Swiss Health Survey were not asked if they were overindebted or had sought debt advice and visited a debt advisory center, they were considered not being overindebted, even though a small proportion of them might meet the criteria and have insufficient household income to meet all payment obligations.

#### Unemployment

Unemployment in both questionnaires was measured by directly asking all survey respondents (overindebtedness survey) or those who were inactive and not currently working (Swiss Health Survey) about their actual (job) situation and about being jobless (involuntarily and registered as such) or not at the time of the survey.

#### Sense of control

Sense of control was measured by a short version of the Pearlin Mastery Scale (33), which assesses belief in having control over the outcomes of events in life. The short scale includes four Likert-scaled items: “When you think about your life, how much do you agree with the following statements?

1. There is really no way I can solve some of the problems I have.
2. Sometimes I feel that I am being pushed around in life.
3. I have little control over the things that happen to me.
4. I often feel helpless in dealing with the problems of life.”

Response categories of the 4-item Pearlin Mastery Scale (PMS-4) ranged from 0 (“strongly agree”) to 3 (“strongly disagree”). Items and their values were summed to obtain a total score between 0 (lowest value) and 12 (highest value). A total score of 0 to 3 was categorized as “no sense of control” (lack of control), and a score of 4 to 7 was categorized as “low sense of control.” A reliability analysis revealed high internal consistency of the four items (Cronbach’s alpha = 0.86).

#### Mental health

Poor mental health was measured by two established and validated multiple-item scales, a short form of the Mental Health Inventory (34) and a subscale (the depression module) of the Patient Health Questionnaire (35).

The 5-item Mental Health Inventory (MHI-5) is a brief, valid, and reliable instrument that is widely used (also in the Swiss Health Survey) to assess mental health or rather to detect mental health problems, particularly mental strain and psychological distress in adults. The MHI-5 covers the following questions that all survey participants, including the clients of the debt advisory centers, were asked: “How often in the previous 4 weeks…

1. Have you been very nervous?
2. Have you felt so down in the dumps that nothing could cheer you up?
3. Have you felt calm, balanced, and relaxed?
4. Have you felt downhearted and depressed?
5. Have you been happy?”

The response scale for all items, which was slightly shortened and adapted for the Swiss Health Survey, ranged from 1 (“always”), 2 (“mostly”), 3 (“sometimes”), 4 (“rarely”), to 5 (“never”). The MHI-5 total score was calculated, first, by recoding reversely directed and formulated items 3 and 5 (1 = 5, 2 = 4, 3 = 3, 4 = 2, 5 = 1), second, by adding up the scores of all five items, and third, by transforming this sum score linearly into a new score on a scale from 0 to 100 (100*[sum score-5]/20). A higher final score indicates low levels of psychological distress and negative feelings (nervous, depressed, uneasy, restless and unbalanced, discouraged, unhappy). A total score from 0 to 52 was considered a high level of psychological distress; a score between 53 and 72 was considered a medium level. When the MHI-5 is dichotomized, a score near or below 60 or exactly at 52 is commonly taken as a cutoff point for the risk group with ‘psychological distress’ (36).

The 9-item Patient Health Questionnaire (PHQ-9) was used to measure symptoms of depression, to diagnose depressive disorders, and to assess depression severity. Survey participants were asked: “Over the last 2 weeks, how often have you been affected by any of the following complaints?

1. Little interest or pleasure in doing things
2. Feeling down, depressed, or hopeless
3. Trouble falling or staying asleep, or sleeping too much
4. Feeling tired or having little energy
5. Poor appetite or overeating
6. Feeling bad about yourself – or that you are a failure or have let yourself or your family down
7. Trouble concentrating on things, such as reading the newspaper or watching television
8. Moving or speaking so slowly that other people could have noticed. Or the opposite – being so fidgety or restless that you have been moving around a lot more than usual
9. Thoughts that you would be better off dead, or of hurting yourself.”

Response categories ranged from 0 (“not at all”), 1 (“on individual days”), 2 (“on more than half the days”), to 3 (“nearly every day”). The PHQ-9 total score was calculated by adding all nine items and their scores, ending up with a final score from 0 to 27. A total score of 15 or higher indicates severe or major depression, and a score from 10 to 14 moderate depression. Cronbach’s alpha as a value for the internal consistency of the 9-item scale was 0.88.

### Analyses

**Figure 2:**
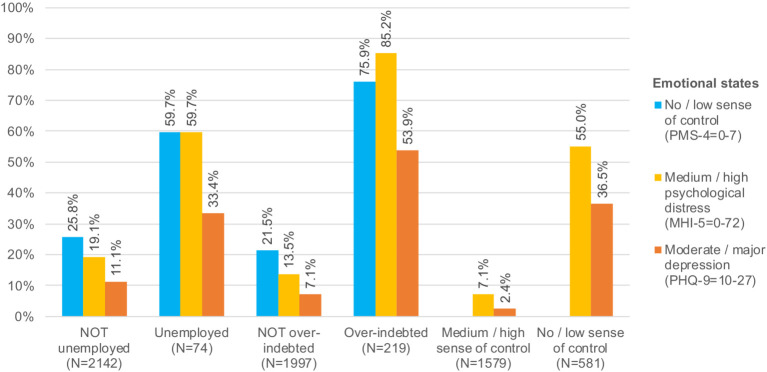
Emotional states and mental health conditions of adult individuals with and without severe loss experiences (loss of job, financial autonomy, and sense of control).

No descriptive statistics for the study population are shown here, as those details have already been provided in a previously published article using the same pooled data set (25). Frequency distributions of the mediator and the two outcome variables were first calculated and stratified by different subgroups of the study population (see Table 1) to gain a first impression of the emotional state and mental health condition of these mutually exclusive subgroups, which are characterized by different combinations of the presence or absence of unemployment and/or overindebtedness. Subsequently, prevalence rates of negative emotional states and poor mental health outcomes, including a non-existent sense of control as a measure of learned helplessness, were calculated, shown, and compared for the unemployed and the NOT unemployed and for the overindebted and the NOT overindebted as well as for participants with and without the sense of control (see Figure 2).

**Table 1:** Health-related characteristics of the study population and specific subgroups (regarding unemployment and overindebtedness).

		Unemployed but not overindebted individuals (*N* = 44)	Overindebted but not unemployed individuals (*N* = 189)	Unemployed and overindebted individuals (*N* = 30)^a^	Neither unemployed nor overindebted individuals (*N* = 1,953)	Total study population (*N* = 2,216)
**Sense of control**(PMS-4)	No (0–3)	7.1%	23.7%	33.3%	2.3%	4.6%
	Low (4–7)	28.6%	49.5%	60.0%	18.9%	22.3%
	Medium (8–10)	40.5%	18.3%	3.3%	39.3%	37.0%
	High (11–12)	23.8%	8.6%	3.3%	39.6%	36.1%
**Psychological distress**(MHI-5)	No/low (73–100)	69.8%	16.6%	3.4%	86.9%	79.6%
	Medium (53–72)	14.0%	17.7%	20.7%	9.6%	10.5%
	High (0–52)	16.3%	65.7%	75.9%	3.5%	9.9%
**Depression**(PHQ-9)	No (0–4)	43.9%	21.4%	6.7%	68.4%	63.0%
	Mild (5–9)	43.9%	25.7%	33.3%	24.6%	25.2%
	Moderate (10–14)	9.8%	28.9%	36.7%	4.7%	7.3%
	Major (15–27)	2.4%	24.1%	23.3%	2.3%	4.5%

^a^The extreme group of doubly exposed: These individuals are jobless or rather involuntarily unemployed AND are seeking advice from one of the official debt advisory centers in the Canton of Zurich.

Bivariate correlation and multivariate regression coefficients were then calculated to uncover the true and independent associations or effects and the direct and indirect paths (see Tables 2, 3) hypothesized in the theoretical path model (see Figure 1). To test for mediation (or confounding), an established and recognized 4-step approach following Baron and Kenny (37) was used as a method or analysis strategy. Table 3 shows the results of the stepwise multiple linear regression analyses.

**Table 2:** Simple correlation matrix of all relevant study variables (scores, mean values, standard deviations, and Pearson’s correlation coefficients *r*).

		Score	M	SD	1	2	3	4	5
1	**Unemployment** (dummy)	0/1	0.03	0.18	–				
2	**Overindebtedness** (dummy)	0/1	0.10	0.30	0.19***	–			
3	**Sense of control** (PMS-4)	0–12	8.9	2.8	−0.15***	−0.41***	–		
4	**Psychological distress** (MHI-5)	0–100	79.2	18.1	−0.19***	−0.57***	0.65***	–	
5	**Depression** (PHQ-9)	0–27	4.6	4.5	0.14***	0.42***	−0.67***	−0.72***	–

*p ≤ 0.05; **p < 0.01; ***p < 0.001; (n.s.) = not significant (*p* > 0.05).

**Table 3:** Stepwise multiple linear regression analyses of associations between studied and assumed exposures, confounder or mediator, and mental health outcomes (regression coefficients, *p*-values).

Outcome variables:	Sense of control			Psychological distress									Depression								
	Step 2			Step 1			Step 3			Step 4			Step 1			Step 3			Step 4		
	B^1)^	*ß* ^2)^	*p*	B^1)^	*ß* ^2)^	*p*	B^1)^	*ß* ^2)^	*p*	B^1)^	*ß* ^2)^	*p*	B^1)^	*ß* ^2)^	*p*	B^1)^	*ß* ^2)^	*p*	B^1)^	*ß* ^2)^	*p*
**Exposure variables:**																					
Unemployment	−0.95 [−1.98]	−0.06 [−0.13]	0.002	−7.69 [−17.2]	−0.08 [−0.17]	<0.001				−4.81 [−9.80]	−0.05 [−0.10]	0.001	1.12 [2.86]	0.05 [0.11]	0.023				0.27	0.01	0.494
Overindebtedness	−3.52 [−3.63]	−0.38 [−0.39]	<0.001	−33.5 [−34.4]	−0.54 [−0.56]	<0.001				−22.5 [−23.0]	−0.37 [−0.38]	<0.001	5.86 [5.98]	0.39 [0.40]	<0.001				2.55	0.17	<0.001
**Confounder / mediator:**																					
Sense of control							4.08	0.63	<0.001	3.14	0.49	<0.001				−1.05	−0.65	<0.001	−0.94	−0.59	<0.001
**Control variables:**
Sex (male)	0.22	0.04	0.044		0.12	<0.001	2.52	0.07	<0.001	3.42	0.10	<0.001		−0.11	<0.001	−0.66	−0.07	<0.001	−0.75	−0.08	<0.001
Age category	0.15	0.09	<0.001		0.09	<0.001	0.95	0.09	<0.001	0.51	0.05	<0.001		−0.11	<0.001	−0.19	−0.07	<0.001	−0.14	−0.06	<0.001
Educational level	0.08	0.06	0.002		0.02	0.278	0.25	0.03	0.065	−0.19	−0.02	0.106		−0.06	0.001	−0.09	−0.05	0.005	−0.04	−0.02	0.202
**R square adjusted**	0.185			0.350			0.438			0.554			0.207			0.461			0.485		
**No. cases in model**	2,150			2,174			2,120			2,120			2,152			2,117			2,117		

^1)^ B = unstandardized regression coefficient.

^2)^ *ß* = standardized regression coefficient (beta coefficient or beta weight).

[] = single effects adjusted only for control variables (explanatory variables not mutually adjusted).

## Results

Unemployed and particularly overindebted individuals not only were much more likely to experience a perceived lack of control or learned helplessness but also had much higher prevalence rates of high psychological distress and major depression than not unemployed or overindebted participants (Figure 2). In turn, a perceived lack of control—because of unemployment and overindebtedness—was accompanied by a remarkably increased likelihood of increased psychological distress or major depression (Figure 2).

Regarding sense of control, level of psychological distress, and severity of depression, unemployed participants—like overindebted participants—differed greatly from those who were not seeking advice from a debt advisory center or who were employed or inactive. What about possible differences in the prevalence of emotional states or mental health conditions between participants who were either unemployed or overindebted and those who were both simultaneously? Approximately 36% of the unemployed (but not overindebted), 73% of the overindebted (but not unemployed), and even 93% of the unemployed *and* overindebted had no or only a low sense of control, whereas only 21% of the others felt that they had no or only little control over outcomes in their lives (Table 1). Approximately one-sixth of the unemployed (and not overindebted), two-thirds of the overindebted (and not unemployed), and more than three-quarters of the unemployed *and* overindebted had high psychological distress compared to less than 4% of the others as the large majority (Table 1). Finally, 12% of the unemployed, 53% of the overindebted, and even 60% of the overindebted unemployed or the unemployed overindebted felt moderately or severely depressed compared to only 7% of the rest of the study population (Table 1).

In sum, unemployment and particularly overindebtedness and the combination of both went along with a significantly reduced sense of control and substantially increased levels of psychological distress and depression. However, overindebtedness seemed to be clearly a stronger risk factor than unemployment in this regard.

Further bivariate statistical analyses for the entire study population confirmed partly or fully some expectations, assumptions, or hypotheses (see Table 2), namely:

- Unemployment and overindebtedness were in fact positively but fairly weakly correlated with each other.
- Unemployment and overindebtedness were negatively and at least partly fairly strongly correlated with perceived sense of control.
- This sense of control, in turn, was very strongly and negatively associated with the two strongly interrelated mental health outcomes under study, psychological distress, and depression.

Finally, multivariate regression analyses following Baron and Kenny’s (35) 4-step approach revealed (see Table 3) the following:

- Exposures (unemployment and overindebtedness) were significantly and positively associated with mental health outcomes (psychological distress and depression) (step 1).
- Exposures were significantly and negatively associated with a third variable (sense of control) as the potential mediator or confounder (step 2).
- This potential mediator or confounder again was very strongly and negatively associated with the outcomes (step 3).
- The original associations between exposures and outcomes fully disappeared or were at least substantially reduced when the potential mediator was taken into account and included in the analysis (step 4).

In sum, stepwise multiple linear regression analyses at least partly confirmed the mediation (or confounding) hypothesis. The total effects (beta coefficients) between exposures and psychological distress as one of two outcomes substantially decreased from −0.08 to −0.05 (unemployment) and from − 0.54 to −0.37 (overindebtedness) when sense of control as the potential mediator or confounder was included in the analysis (Table 3). A similar pattern was found for the second outcome, depression: Total effects decreased from 0.05 to 0.01 or from 0.39 to 0.17, respectively. Indirect paths or effects via sense of control as the mediator or confounder (steps 2 and 4) turned out to be similarly strong or even stronger than direct effects (step 4) at least in the prediction of depression. Indirect effects calculated either by the difference or the product of the beta coefficients were quite substantial for at least one exposure (overindebtedness) and both outcomes (psychological distress/depression): *ß* = −0.03 / 0.04 (unemployment) and *ß* = −0.17 / 0.22 (overindebtedness).

However, only partial and not complete mediation or confounding was found for the associations under study, as direct effects in the fully specified and adjusted model (step 4) did not completely disappear but remained, at least in the case of overindebtedness. In other words, as expected, a significant part of the negative mental health effects of unemployment and overindebtedness was presumably mediated or confounded by the resulting feeling of having no or only low control over outcomes in life. A substantial part of the effects remained even in the fully adjusted model, i.e., after adjustment for one another and different control variables. A perceived lack or decreased sense of control—as a result of life-changing experiences and critical life events beyond one’s control—was found to be the strongest predictor of a high level of psychological distress and major depression, closely followed by overindebtedness. Contrary to such constant conditions, unemployment as a temporary condition had no effect or only a comparatively low independent effect on mental health.

As Table 3 shows, the three considered risk and influence factors (unemployment, overindebtedness, and perceived sense of control) together explained a large part of the statistical variance of the mental health outcomes, namely, 55% of the psychological distress measure (MHI-5) and 49% of the depression scale (PHQ-9).

However, in fully adjusted and specified multiple linear regression analyses that included both main predictors (unemployment and overindebtedness) and the potential mediator or confounder (sense of control), associations or beta coefficients as the effect measures between predictors and mental health outcomes were substantially reduced but still persisting and significant, except for unemployment and depression (see step 4 in Table 3).

## Discussion

In contrast to our own previous study (1), which was mainly focused on potential differences between overindebted individuals and the general population regarding socio-demographic and economic characteristics and particularly traditional debt parameters (amount of debt, duration and level of indebtedness, etc.) and on the effects of these debt parameters on the mental health outcomes of overindebted individuals as the study population, the present follow-up study has widened and shifted its research focus and introduced unemployment as a second potentially important predictor of a low sense of control and poor mental health. This study not only looked at overindebted individuals but also at the general population as a whole when examining causes of poor mental health and particularly when studying associations between a sense of control or mastery and mental health outcomes. Furthermore, a low sense of control in this study was considered a potential mediator or confounder, i.e., an explanatory factor that presumably is closely related to critical life events and experiences of unemployment and overindebtedness, whereas in the previous study, mastery was simply introduced as a health-protective factor and ‘control’ variable, specifically for overindebted individuals in poor health and “learned helplessness” (1).

The main findings of this study largely confirm the hypothesized associations and assumed causal paths shown in Figure 1 and can be summarized as follows:

- Unemployed and particularly overindebted individuals, and especially those who combine both conditions, are much more likely to have no or only a low sense of control, to have a high level of psychological distress, and to feel moderately or even severely depressed.
- The strong association found between overindebtedness and depression is substantially mediated or confounded by the perceived loss or lack of control over life. However, to a lesser extent, this mediation or confounding also applies to the very strong association between overindebtedness and psychological distress. In turn, unemployment is only weakly associated with the two mental health outcomes when overindebtedness (as a possible consequence of long-term unemployment) is additionally considered and included in the regression model.

The finding that unemployment and overindebtedness both go along with an increased likelihood of a generalized feeling of loss of control but to very different degrees may be attributable to the fact that the two life events are not critical and harmful to the same extent, at least not in Switzerland. Whereas unemployment is usually a single, temporary, socially accepted, and financially supported or insured event that has occurred for defined reasons, overindebtedness is usually the result of several unfavorable or unfortunate circumstances and events and the culmination of long-lasting social and financial misery and hardship. Overindebtedness is, in general, much more socially tabooed and stigmatized than unemployment. In other words, overindebted individuals have often already experienced several personal crises (unemployment, business failure, working poverty, social welfare, disease, divorce, addiction problems, etc.), whereas unemployed persons mostly face a single crisis and are economically secured for quite a while by unemployment insurance. In contrast to several partly unexpected and uncontrollable personal crises leading to overindebtedness and creating a feeling of losing control over events in one’s life, a single crisis like unemployment presumably does not immediately result in a shaken sense of control.

Unlike unemployment, overindebtedness was found to be a strong risk factor for psychological distress and depression. The effect of overindebtedness on the two mental health outcomes turned out to be partly or mainly indirect and significantly mediated or confounded by a lack or low sense of control. This sense of control was found to be a strong correlate of overindebtedness and simultaneously proved to be the strongest of all the studied predictors of poor mental health.

Unemployment, in contrast, was not a particularly strong correlate of overindebtedness and was not an important predictor of poor mental health. This might be because the overindebtedness survey of 2019 had not assessed the duration of unemployment, which therefore could not be taken into account in this study. It seems plausible that longer lasting unemployment would have been much more strongly related to overindebtedness and poor mental health, whereas short-term unemployment presumably does not significantly increase the risk of overindebtedness and poor mental health. Previous studies confirm this assumption by showing that particularly long-term unemployment is associated with poor mental health, suicide, and higher levels of distress (2, 15).

Another indication that long-term unemployment presumably quite often ends up in overindebtedness is the fact that overindebted individuals in this study and study population most frequently named unemployment as one of the main reasons for their indebtedness (38%). Overindebtedness can thus be viewed as a direct result, or the tip of the iceberg, of long-lasting unemployment and subsequently being dependent on social welfare. This is probably why most of the single and total effects of unemployment on the mental health outcomes examined in this study disappeared when overindebtedness was included as a second exposure variable in the regression model, but not the other way around (see beta coefficients in square brackets in Table 3).

In recent years, many and mostly Scandinavian or Central European studies have investigated either the association between unemployment and mental health (2, 4, 9, 11, 18, 23, 29) or between overindebtedness and mental health (3, 5, 12, 21, 25–28). Only very few studies in the research literature have examined both relationships (3) or perceived sense of control as a potential mediator in one of these relationships (17, 25). And up to now, no other study has been published, particularly not for Switzerland, that has investigated both of these potential risk factors for mental health and additionally considered perceived sense of control as a potential mediator.

However, despite the lack of similar and completely comparable studies, this study supports and confirms the individual findings of previous international studies in Sweden, Finland, Germany, and Portugal. Some specific results of the present study are fully in line with a recent study in Portugal (17) with 236 overindebted and 129 non-overindebted Portuguese consumers, which found that overindebtedness is associated with lower levels of perceived control and that this sense of control partly mediates the negative impact of overindebtedness on health and wellbeing.

What is new in this study and has not been found before is that unemployment no longer seems to play a key role as a risk factor for poor mental health when both overindebtedness and sense of control are considered and included in the analysis. In other words, unemployed people do not seem to show poor mental health as long as they are not overindebted and do not feel a loss of control. This makes sense, as only long-term unemployment is expected to result in overindebtedness and a low sense of control.

### Strengths and limitations

This study fills a research gap in an unexplored or at least under-explored part of the population. Relationships or associations between unemployment, overindebtedness, sense of control, and poor mental health have not been studied before or together, especially not in Switzerland, where overindebtedness and unemployment are comparably rare and particularly stigmatized phenomena and hence are largely ignored in research. This applies particularly to the potential key role of sense of control in the relationship between unemployment and poor mental health, which has not been previously examined in a population-based sample and with additional consideration of overindebtedness as a possible consequence of long-term unemployment (and/or poor mental health).

Another advantage is the unique and fairly large study sample and the use of pooled survey data that includes an oversampled proportion of overindebted individuals who are usually (expected to be) strongly underrepresented in population-based surveys and studies, as are also unemployed or all socially deprived or disadvantaged groups. Only such pooled data with two cross-sectional surveys merged together and two linked subsamples—similar to a case–control study—provided enough statistical power, sufficiently large numbers of overindebted and/or unemployed individuals, and a comparison with the general population to be able to conduct multivariate association analyses using inferential statistics and to obtain statistically significant effects between exposures and outcomes.

The study nevertheless has limitations. First, cross-sectional survey data were used for this study, which do not allow us to test for causality. It remains unclear in the end if poor mental health is more a cause or a consequence of unemployment and/or overindebtedness and if it occurs already before or only after the two conditions and critical life events. In addition, the data do not allow assessment of long-term unemployment as a possible precondition of overindebtedness and proven risk factor for mental health, nor do differentiate it from short-term unemployment as arguably unproblematic regarding poor mental health. The duration of unemployment was not captured directly or—in a cross-sectional study—recorded indirectly by asking about current activity or employment status at several points in time.

Second, a systematic error or misclassification bias cannot be excluded, as the survey participants were not asked directly if they were overindebted; they were selected and classified as overindebted simply by the fact that they were clients of an official debt advisory center seeking advice from debt advisers. Asking such delicate questions directly would have been problematic, with the risk of missing answers, refusal of survey participation, early breaking off of the interview, or not completing the questionnaire. Treating or assessing the entire sample of the Swiss Health Survey as not overindebted due to unavailable related information is also not completely unproblematic. Overindebtedness in this random sample of the general population is most likely underestimated, as an estimated 6 to 8% of the resident population in Switzerland is presumably overindebted (1), even though overindebtedness might generally be underreported in population surveys due to non-responding (missing answers) or non-participation (self-exclusion from survey participation) of overindebted individuals. However, as this potential misclassification between the exposed and the non-exposed (here: overindebted or not) is unrelated to the occurrence or presence of illness (here: psychological distress and depression) and therefore non-differential, the possible bias tends to minimize and underestimate the true association. This means that such misclassification does not question the results of the study. On the contrary, without such misclassification, the associations found would have been even stronger than they are now.

## Conclusion

The very poor mental health of unemployed and particularly overindebted individuals emerges as largely influenced and explained—directly and indirectly—by a person’s sense of control. Therefore, and as (long-term) unemployment and overindebtedness often have structural and not (only) individual causes, and insofar cannot be avoided, measures to prevent mental health problems in unemployed and/or overindebted persons should not be restricted to job placement and employment services or to debt advisory services. They should, in addition, focus on strengthening the affected persons’ sense of control and empowering them. Sense of control can be viewed thereby as a learned and quite stable but basically modifiable general belief that one can and does master, control, and shape one’s own life. Somehow strengthening one’s sense of control may be a potentially successful strategy for coping with all health-threatening critical life events and conditions.

## Data availability statement

Publicly available data were analyzed in this study. Swiss Health Survey data are available from the Swiss Federal Office of Statistics for scientific purposes only, for a fee and on the basis of a written contract and declared research topic(s).

## Ethics statement

The study did not require ethics approval because all self-reported survey data used in this study were voluntarily and anonymously collected and therefore did not fall within the scope of the Swiss Federal Act on Research Involving Human Beings which does not apply to such anonymously collected or subsequently anonymised health-related data. The studies were conducted in accordance with the local legislation and institutional requirements. Written informed consent for participation was not required from the participants or the participants’ legal guardians/next of kin because use of completely anonymous self-reported secondary survey data which are publicly accessible (in the case of the Swiss Health Survey).

## Author contributions

OH: Conceptualization, Data curation, Formal analysis, Investigation, Methodology, Supervision, Writing – original draft, Writing – review & editing.
